# Rno-microRNA-30c-5p promotes myocardial ischemia reperfusion injury in rats through activating NF-κB pathway and targeting SIRT1

**DOI:** 10.1186/s12872-020-01520-2

**Published:** 2020-05-20

**Authors:** Jianfeng Chen, Mingming Zhang, Shouyan Zhang, Junlong Wu, Shufeng Xue

**Affiliations:** 1 Department of Cardiology, Luoyang Central Hospital Affiliated to Zhengzhou University, No. 288, Zhongzhou Middle Road, Luoyang City, 471000 Henan Province China; 2 Department of Orthopedics, Luoyang Central Hospital Affiliated to Zhengzhou University, No. 288, Zhongzhou Middle Road, Luoyang City, 471000 Henan Province China

**Keywords:** Myocardial ischemia reperfusion injury, Rno-miR-30c-5p, Inflammation, Apoptosis, SIRT1, NF-κB pathway

## Abstract

**Background:**

This study aimed to investigate the regulatory effect of rno-microRNA-30c-5p (rno-miR-30c-5p) on myocardial ischemia reperfusion (IR) injury in rats and the underlying molecular mechanisms.

**Methods:**

A rat model of myocardial IR injury was established. The infarct size was detected by 2,3,5-triphenyltetrazolium chloride staining. The pathologic changes of myocardial tissues were detected by hematoxylin-eosin staining. The apoptosis of myocardial cells was measured by TUNEL staining and flow cytometry. The mRNA expression of rno-miR-30c-5p and Sirtuin 1 (SIRT1) was detected by quantitative real-time PCR. The levels of IL-1β, IL-6 and TNF-α were detected by enzyme linked immunosorbent assay. The protein expression of Bax, Bcl-2, caspase-3, p-IκBα, IκBα, p-NF-κB p65, NF-κB p65 and SIRT1 was detected by Western blot. The interaction between rno-miR-30c-5p and SIRT1 was predicted by TargetScan, and further identified by dual luciferase reporter gene and RNA immunoprecipitation assay.

**Results:**

The myocardial IR injury model was successfully established in rats. IR induced the myocardial injury in rats and increased the expression of rno-miR-30c-5p. Overexpression of rno-miR-30c-5p enhanced the inflammation, promoted the apoptosis, and activated NF-κB pathway in IR myocardial cells. SIRT1 was the target gene of rno-miR-30c-5p. Silencing of SIRT1 reversed the effects of rno-miR-30c-5p inhibitor on the apoptosis and NF-κB pathway in IR myocardial cells.

**Conclusions:**

Rno-miR-30c-5p promoted the myocardial IR injury in rats through activating NF-κB pathway and down-regulating SIRT1.

## Background

Ischemic heart disease is a series of diseases characterized by myocardial ischemia, such as angina pectoris and myocardial infarction [1]. Recently, reperfusion of the ischemic myocardium is one of the most common therapeutic strategies for ischemic heart diseases [2]. Although restoring blood flow in time can relieve myocardial infarction to a great extent, the prognosis of patients remains poor due to the ischemia reperfusion (IR) injury on myocardium [3]. Therefore, it is urgent to find out novel therapeutic methods and targets for myocardial IR injury.

The genome-wide investigations of genetic variants, epigenetic modifications, and gene expression profiles optimize the search for novel diagnostic or therapeutic targets for IR injury in the post-genomic era [4]. MicroRNAs (miRNAs) are a class of small endogenous noncoding RNAs with 19–25 nucleotides in length, which modulate gene expression at the post-transcriptional level [5, 6]. A systematic comparison of IR injury-induced miRNA expression changes in rats identifies several potential cardioprotective miRNA targets (protectomiRs), including Rno-miR-125b*, − 139-3p, − 320, − 532-3p, and − 188 [7]. By using bioinformatics methods based on topological or network dynamical approaches, the mRNA targets of protectomiRs can be predicated. Nevertheless, all unbiased omics approaches and their bioinformatic evaluation need to be verified by rigorous experimental validation at the transcript and protein levels [8].

Recently, studies have indicated that miRNAs play important regulatory roles in myocardial IR injury [9]. Yuan et al. [10] have proved that the inhibition of rno-miR-181b-5p protects cardiomyocytes against I/R injury through targeting AKT3 and PI3KR3. Zhao et al. [11] have reported that mmu-miR-374a protects against myocardial IR injury in mice via targeting MAPK6 pathway. Song et al. [12] have indicated that rno-miR-30b overexpression has anti-apoptotic effect on cardiomyocytes at early phase of myocardial IR injury in a rat model. MiR-30c-5p is another subtype of miR-30 that also involved in the process of IR injury. Zhou et al. [13] have proved that rno-miR-30c-5p is a potential diagnostic marker for I/R-induced kidney injury in rats. Li et al. [14] have shown that hydrogen sulfide protects spinal cord and induces autophagy in a rat model of spinal cord IR injury via regulating rno-miR-30c-5p. However, the regulatory effect and mechanism of rno-miR-30c-5p on myocardial IR injury remain unclear.

Nuclear factor κB (NF-κB) is involved in the regulation of multiple biological functions including innate immunity, inflammation, cell proliferation and apoptosis [15, 16]. Accumulating researches have revealed that myocardial IR injury is associated with the activation of NF-κB [17]. In addition, emerging evidence has indicated that miRNAs play vital roles in myocardial IR injury by regulating NF-κB pathway. For instance, mmu-miR-146a overexpression reduces myocardial IR injury via inhibiting the activation of NF-κB pathway [18]. However, whether the regulatory effect of rno-miR-30c-5p on myocardial IR injury is involved in NF-κB pathway is unknown.

In this study, we explored the regulatory effect of rno-miR-30c-5p on myocardial IR injury in rats, as well as the underlying molecular mechanisms. Our results indicated that rno-miR-30c-5p promoted the myocardial IR injury in rats through activating NF-κB pathway and down-regulating SIRT1. Our findings may provide a new theoretical foundation for the treatment of myocardial IR injury in clinical practice.

## Methods

### Animals

Male Sprague-Dawley (SD) rats (weighting 180–200 g) were provided by Peking University Laboratory Animal Center. All rats were kept at 22–24 °C and 55–60% humidity on a 12 h light-dark cycle with free access to water and food. At the end of the study, all rats (220-270 g) were anesthetized by an intraperitoneal injection of 50 mg/kg pentobarbital sodium, and then sacrificed by cervical dislocation. All animal experiments were conducted strictly in accordance with the National Institutes of Health guide for the care and use of Laboratory animals.

### Establishment of the myocardial IR model in rats

Rats weighing 200-240 g were used to establish the IR model. Briefly, rats were anesthetized with pentobarbital sodium (50 mg/kg, i.p.). The left anterior descending coronary artery (LAD) was ligated using 6–0 silk suture slipknot for 30 min, and then reperfused for 2 h. Myocardial ischemia was confirmed by the appearance of regional epicardial cyanosis over the myocardial surface and by arrhythmia. Successful reperfusion was confirmed by the disappearance of epicardial cyanosis and the production of epicardial hyperemia and arrhythmia (IR group). Rats undergoing thoracotomy without LAD ligation were considered as the Sham group.

### Hemodynamic examination

One week after modeling, the hemodynamic parameters including left ventricular ejection fraction (LVEF), left ventricular systolic pressure (LVSP), left ventricular end-diastolic volume (LVEDV), left ventricular end-systolic volume (LVESV), left ventricular end-diastolic pressure (LVEDP), the maximum up rate of left ventricular pressure (+dP/dt_max_), and the maximum down rate of left ventricular pressure (−dP/dt_max_) were measured using a Vevo770 scanner (VisualSonics, Toronto, Canada).

### Infarct size measurement

The infarct size was detected using 2,3,5-triphenyltetrazolium chloride (TTC) (Sangon, Shanghai, China) staining. Briefly, the ventricle was sliced into pieces with equal thickness. The slices were then incubated in 2% TTC for 15 min in the dark and fixed in 10% formaldehyde for 10 min. The infarct area was measured by an image analyzer. The infarct size was calculated as the ratio of the infarct area and total area (%).

### Hematoxylin-eosin (HE) staining

The ventricle was fixed in 4% formaldehyde overnight at 4 °C. Followed by dehydration, vitrification, and paraffin-embedding, the tissue samples were cut into 5 μm-thick slices. The sections were then deparaffined in xylene, rehydrated in gradient ethanol, and stained with hematoxylin for 4 min and Eosin for 2 min. The histopathological changes were observed under a light microscope (400 ×).

### Terminal dexynucleotidyl transferase-mediated dUTP nick end labeling (TUNEL) staining

Cell apoptosis was detected using a TUNEL kit (Beyotime, Shanghai, China). Briefly, the paraffin-embedded tissue sections were deparaffined in xylene, and rehydrated in gradient ethanol. The sections were then incubated with DNase-free Proteinase K for 20 min, with 3% hydrogen peroxide (in PBS) for 10 min, and with TUNEL mix for 60 min. After 30 min of incubation with Streptavidin-HRP, the apoptotic cells were visualized using diaminobenzidine, and re-stained with hematoxylin. The apoptotic cells were counted under a light microscope (400 ×) at five randomly selected fields.

### Isolation of IR myocardial cells

The myocardial tissues at the ischemic site were collected and homogenated. The tissue homogenate was digested with collagenase IV (0.45 mg/ml) containing 0.1% trypsin and 15 μg/ml DNase I. After centrifugation, the residue (myocardial cells) was collected. Myocardial cells were cultured in RPMI 1640 medium (Gibco, USA) containing 15% FBS, and maintained in an incubator at 37 °C with 5% CO_2_.

### Cell transfection and grouping

The rno-miR-30c-5p mimics, rno-miR-30c-5p inhibitor, SIRT1 siRNA1–3 and the negative controls (mimics NC, inhibitor NC and si-NC) were purchased from Genepharma (Shanghai, China). IR myocardial cells were seeded into 24-well plates (1 × 10^5^/well), and cultured until 80% confluence. Cells were then transfected with the above agents using Lipofectamine 3000. IR myocardial cells were randomly divided into 9 groups: IR (no treatment), inhibitor NC, rno-miR-30c-5p inhibitor, mimics NC, rno-miR-30c-5p mimics, si-NC + inhibitor NC, siRNA2 + inhibitor NC, siRNA2 + rno-miR-30c-5p inhibitor, and si-NC + rno-miR-30c-5p inhibitor group. After 48 h of transfection, cells were used for subsequent experiments.

### Flow cytometry

Myocardial cells were washed with PBS twice and then stained with Annexin V-fluorescein isothiocyanate (FITC) and propidium iodide (PI) for 15 min in the dark. The apoptosis was detected by a flow cytometer (Beckman Coulter, USA).

### Enzyme linked immunosorbent assay (ELISA)

The myocardial cells and tissues were homogenated and maintained on ice. The levels of inflammatory factors including TNF-α, IL-1β and IL-6 were detected using specific ELISA kits (Thermo Fisher Scientific, USA) in accordance with the manufacturer’s instructions.

### Quantitative real-time PCR

Total RNA was extracted from myocardial cells and tissues using TRIZOL (Invitrogen, USA). Total RNA was then reverse-transcribed into cDNA using a Reverse Transcription Kit (Thermo Fisher Scientific, USA). qRT-PCR was performed on a PCR instrument (Bio-Rad, USA) using SYBR Green Mixture (Roche, Switzerland). Primers were shown as follows: rno-miR-30c-5p F: 5′-GGGGTGTAAACATCCTACAC-3′, R: 5′-GTGGAGTCGGCAATTGCACT-3′; U6 F: 5′-GCTTCGGCAGCACATATACTAAAAT-3′, R: 5′-CGCTTCAC GAATTTG CGTGTCAT-3′; SIRT1 F: 5′-AAGGAGCAGATTAGTAAGC-3′, R: 5′-TAGAGGATAAGGCGTCAT-3′; GAPDH F: 5′-GACGGCCGCATCTTCTTGT-3′, R: 5′-CACACCGACCTTCACCATTTT-3′. GAPDH and U6 with stable expression were used as internal controls of SIRT1 and rno-miR-30c-5p, respectively.

### Western blot

Total protein was extracted from myocardial cells and tissues using RIPA lysis buffer (Beyotime, Shanghai, China). The protein samples (50 μg) were separated by 10% SDS-PAGE and then transferred onto polyvinylidenedifluoride membrane. After blocked with 5% skim milk in TBST for 2 h, the membrane was incubated with specific primary antibody (anti-Bax, 1:1000, 14,796; anti-Bcl-2, 1:1000, 4228 s; anti-IκBα, 1:500, #4814; anti-p-IκBα, 1:500, #2859; anti-SIRT1, 1:1000, #2310, Cell signal, USA; anti-NF-κB p65, 1:1000, SAB4502610; anti-p-NF-κB p65, 1:1000, SAB4301496, Sigma Aldrich, USA; anti-caspase-3, 1:1000, sc-271,759; anti-β-actin, 1:1000, sc-517,582, Santa Cruz, USA) overnight at 4 °C. After washed with TBST for three times, the membrane was incubated with horseradish peroxidase (HRP)-labeled secondary antibody for 2 h at 25 °C. The protein bands were visualized using a HRP kit and quantified by an ECL system (Thermo Fisher Scientific, USA).

### TargetScan prediction

The targets of rno-miR-30c-5p were predicted using TargetScan 7.1 (http://www.targetscan.org/vert_71/). A total of 1249 transcripts containing 1835 sites were predicted (Table S1). A target gene SIRT1 (ENST00000212015.6) was selected due to its important role in myocardial IR injury (Table S2).

### Dual luciferase reporter gene (DLR) assay

DLR assay was used to identify the targeting relationship between SIRT1 and rno-miR-30c-5p. The fragment of SIRT1, containing the binding site was amplified and cloned into pmirGLO luciferase vector (Promega, USA) to construct wild pmirGLO-WT-SIRT1–3′-UTR (SIRT1-Wt) and mutant pmirGLO-MUT-SIRT1–3′-UTR (SIRT1-Mt). Myocardial cells were co-transfected with SIRT1-Wt/Mt and rno-miR-30c-5p mimics/mimics NC using Lipofectamine 3000. Myocardial cells were randomly divided into 4 groups: SIRT1-Mt + rno-miR-30c-5p mimics, SIRT1-Mt + mimics NC, SIRT1-Wt + rno-miR-30c-5p mimics, and SIRT1-Wt + mimics NC group. After 48 h of transfection, the luciferase activity was measured using a dual luciferase kit (Promega).

### RNA immunoprecipitation (RIP) assay

RIP assay was performed using a Magna RIP Kit (Millipore, USA). Briefly, myocardial cells were lysed in lysis buffer. The cell lysate was then incubated with anti-Ago2 or IgG-coated beads at 4 °C for 2 h. After washed with PBS, the RNA-protein-beads complexes were isolated using Trizol. The expression of rno-miR-30c-5p and SIRT1 was measured by qRT-PCR.

### Statistical analysis

Three independent repetitions were conducted for each sample. Data were expressed as mean ± standard deviation (SD), and analyzed using SPSS 22.0 Statistical Software (Chicago, IL). Differences among multi-groups were analyzed by one-way ANOVA followed by Tukey’s post hoc test. Differences between two groups were analyzed by Student’s t test. The level of statistical significance was set at *p* < 0.05.

## Results

### IR induces myocardial injury in rats

**Fig. 1:**
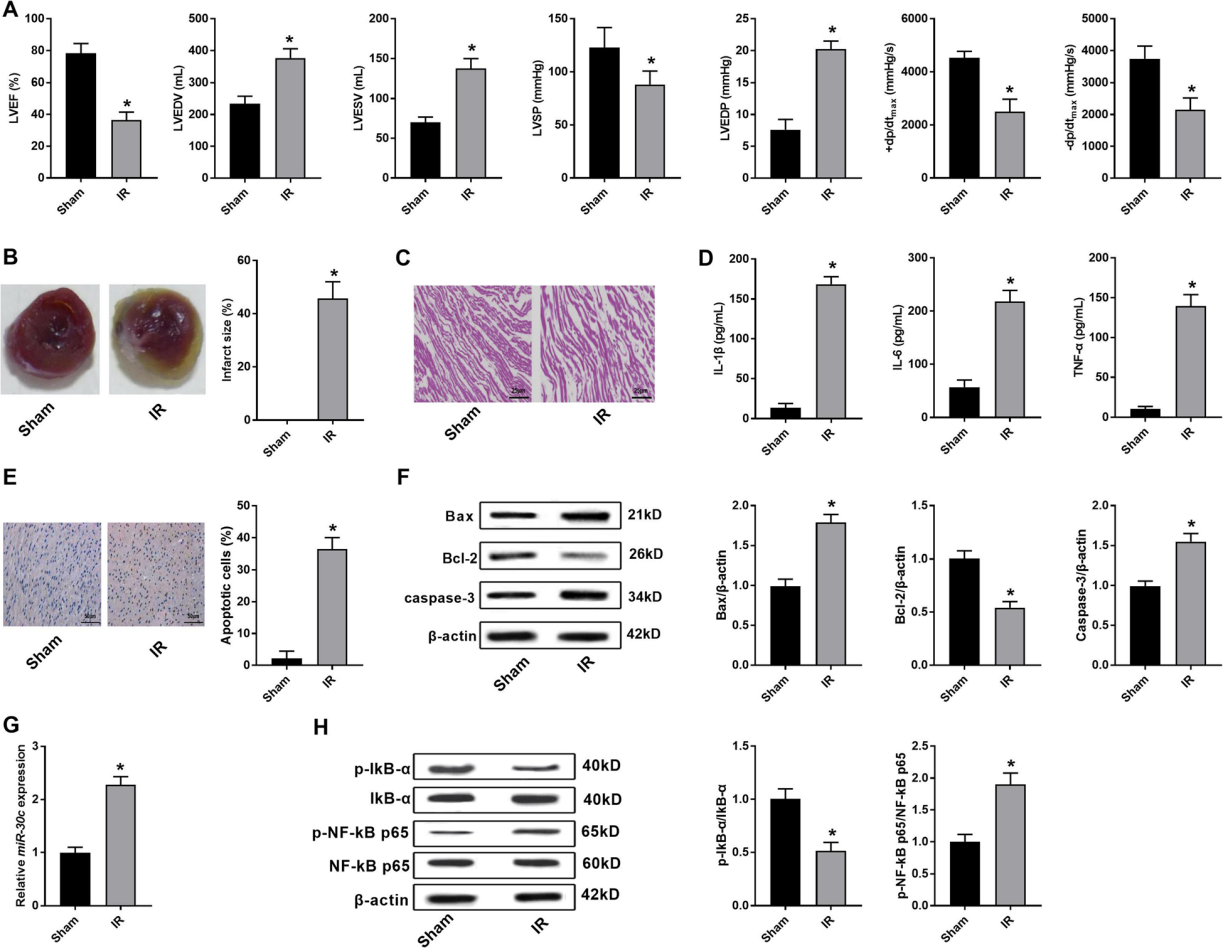
IR induced myocardial injury in rats. **a** The levels of LVEF, LVEDV, LVESV, LVSP, LVEDP, +dP/dt_max_ and -dP/dt_max_ (*N* = 25 each group). **b** Infarct size (*N* = 10 each group). **c** HE staining of myocardial tissues (× 400, *N* = 5 each group, representative images were shown). **d** The levels of IL-1β, IL-6 and TNF-α were detected by ELISA (*N* = 10 each group). **e** The apoptotic cells (%) were measured by TUNEL staining (*N* = 5 each group, representative images were shown). **f** The expression of Bax, Bcl-2 and caspase-3 was detected by Western blot (*N* = 5 each group, representative images were shown). **g** The expression of rno-miR-30c-5p was detected by qRT-PCR (*N* = 5 each group). **h** The expression of p-IκBα, IκBα, p-NF-κB p65 and NF-κB p65 was detected by Western blot (*N* = 5 each group, representative images were shown). Data were presented as mean ± SD (three independent repetitions for each sample). ^*^P < 0.05, vs. Sham group

As shown in Fig. 1a, the levels of LVEF, LVSP, +dP/dt_max_ and -dP/dt_max_ were significantly lower, and the levels of LVEDV, LVESV and LVEDP were significantly higher in the IR group than those in the Sham group (*P* < 0.05). The infarct size was significantly higher in the IR group than that in the Sham group (*P* < 0.05) (Fig. 1b). HE staining showed that the myocardial fibers in the Sham group were orderly arranged without inflammatory cell infiltration. Disorganized myocardial fibers accompanied with obvious inflammatory cell infiltration were observed in the IR group (Fig. 1c). The levels of IL-6, IL-1β and TNF-α in the Sham group were significantly higher than those in the IR group (*P* < 0.05) (Fig. 1d). In addition, TUNEL assay showed that IR significantly promoted the apoptosis of myocardial cells (*P* < 0.05) (Fig. 1e). The protein expression of Bax, caspase-3, and p-NF-κB p65/NF-κB p65 was significantly increased, and the protein expression of Bcl-2 and p-IκBα/IκBα was significantly decreased in the IR group compared with that in the Sham group (*P* < 0.05) (Fig. 1f and h). Note worthily, the expression of rno-miR-30c-5p was significantly higher in the IR group than that in the Sham group (*P* < 0.05) (Fig. 1g). All these results suggested that IR could induce the myocardial injury in rats.

### Rno-miR-30c-5p enhances the inflammation, promotes the apoptosis, and activated NF-κB pathway in IR myocardial cells

**Fig. 2:**
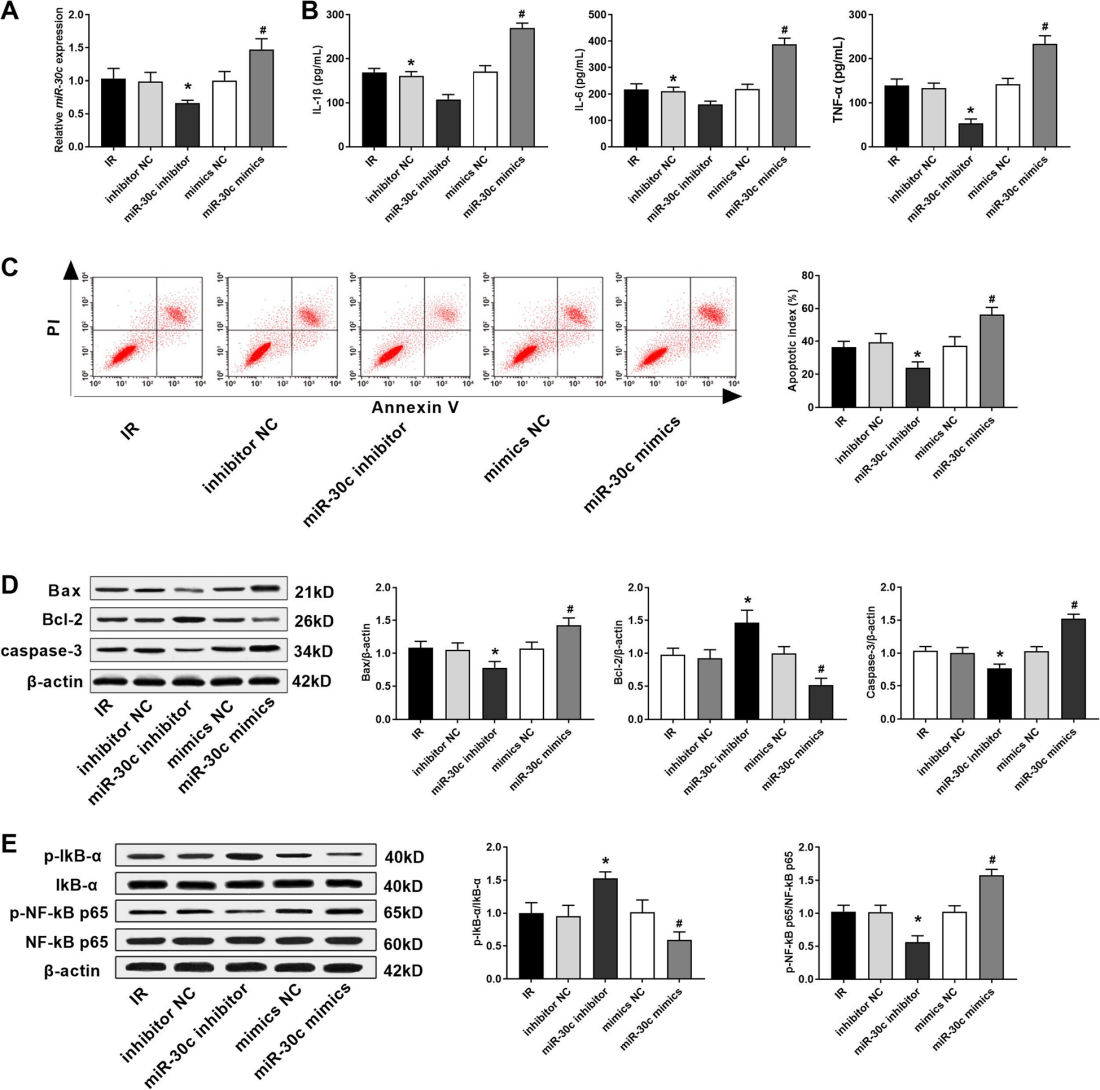
Rno-miR-30c-5p enhanced the inflammation, promoted the apoptosis, and activated NF-κB pathway in IR myocardial cells. **a** The mRNA expression of rno-miR-30c-5p was detected by qRT-PCR (*N* = 3 each group). **b** The levels of IL-1β, IL-6 and TNF-α were detected by ELISA (*N* = 3 each group). **c** The apoptotic index (%) was detected by Flow cytometry (*N* = 3 each group). **d** The expression of Bax, Bcl-2 and caspase-3 was detected by Western blot (*N* = 3 each group). **e** The expression of p-IκBα, IκBα, p-NF-κB p65 and NF-κB p65 was detected by Western blot (*N* = 3 each group). Data were presented as mean ± SD (three independent repetitions for each sample). ^*^P < 0.05, vs. IR and inhibitor NC group; ^#^*P* < 0.05, vs. IR and mimics NC group

As shown in Fig. 2a, the expression of rno-miR-30c-5p in IR myocardial cells was significantly decreased in the rno-miR-30c-5p inhibitor group, and increased in the rno-miR-30c-5p mimics group compared with the IR group (*P* < 0.05). The expression of rno-miR-30c-5p was not significantly influenced by the transfection of either inhibitor NC or mimics NC (Fig. 2a). The levels of IL-6, IL-1β and TNF-α were significantly decreased in the rno-miR-30c-5p inhibitor group, and were significantly increased in the rno-miR-30c-5p mimics group compared with those in the IR group (*P* < 0.05) (Fig. 2b). The apoptotic index was significantly lower in the ron-miR-30c-5p inhibitor group and was significantly higher in the rno-miR-30c-5p mimics group than that in the IR group (*P* < 0.05) (Fig. 2c). In addition, the transfection of rno-miR-30c-5p inhibitor significantly decreased the protein expression of Bax, caspase-3 and p-NF-κB p65/NF-κB p65, and increased the protein expression of Bcl-2 and p-IκBα/IκBα in IR myocardial cells. The effect of rno-miR-30c-5p mimics on the expression of the above proteins was opposite to that of rno-miR-30c-5p inhibitor (*P* < 0.05) (Fig. 2d and e). These results indicated that rno-miR-30c-5p might enhance the inflammation, promote the apoptosis and activate NF-κB pathway in IR myocardial cells.

### SIRT1 is the target gene of rno-miR-30c-5p

**Fig. 3:**
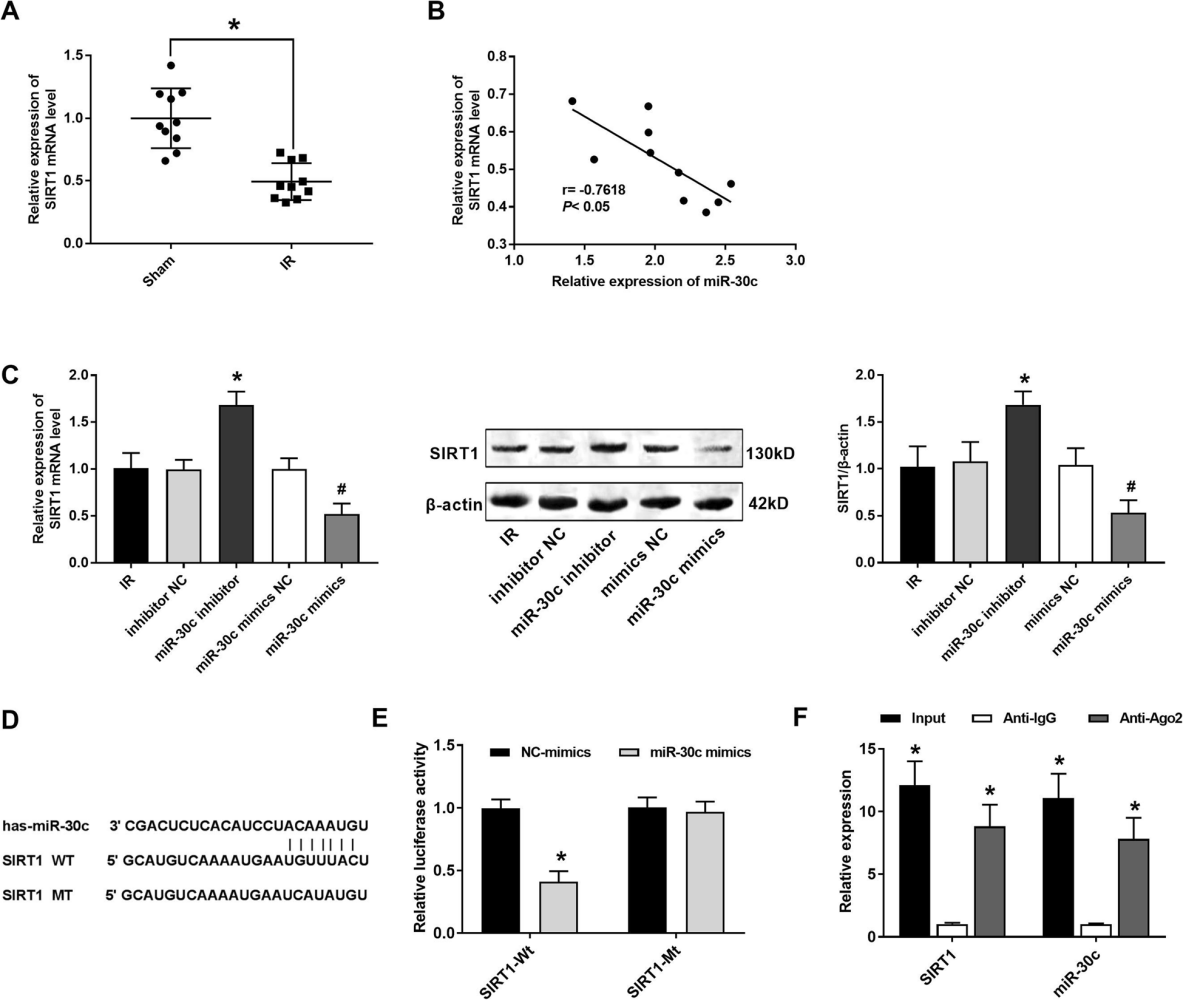
SIRT1 was the target gene of rno-miR-30c-5p. **a** The expression of rno-miR-30c-5p was detected by qRT-PCR (*N* = 5 each group). **b** Correlation analysis between the expression of rno-miR-30c-5p and SIRT1. **c** The mRNA and protein expression of SIRT1 were detected by qRT-PCR and Western blot (*N* = 3 each group). **d** The binding site of rno-miR-30c-5p at 3′-UTR of SIRT1 was predicted by TargetScan software. **e** The interaction between rno-miR-30c-5p and SIRT1 was analyzed by DLR assay. **f** The interaction between rno-miR-30c-5p and SIRT1 was analyzed by RIP assay. Data were presented as mean ± SD (three independent repetitions for each sample). ^*^P < 0.05, vs. Sham group (**a**); ^*^P < 0.05, vs. IR and inhibitor NC group, ^#^*P* < 0.05, vs. IR and mimics NC group (**c**); ^*^P < 0.05, vs. NC-mimics group (**e**); ^*^P < 0.05, vs. Anti-IgG group (**f**)

As shown in Fig. 3a, the expression of SIRT1 in the IR group was significantly lower than that in the Sham group (*P* < 0.05). The expression of rno-miR-30c-5p was negatively correlated with the expression of SIRT1 (*P* < 0.05) (Fig. 3b). The transfection of rno-miR-30c-5p inhibitor and rno-miR-30c-5p mimics significantly increased and decreased the expression of SIRT1 in IR myocardial cells at the mRNA and protein level, respectively (*P* < 0.05) (Fig. 3c). A binding site at 3′-UTR of SIRT1 was predicted on rno-miR-30c-5p by TargetScan (Fig. 3d). DLR assay showed that the luciferase activity was significantly reduced in the SIRT1-Wt + rno-miR-30c-5p mimics group compared with that in the SIRT1-Wt + NC-mimics group (*P* < 0.05) (Fig. 3e). RIP assay further indicated the expression of SIRT1 and rno-miR-30c-5p was significantly decreased in the Anti-IgG group compared with that in the Input group (*P* < 0.05) (Fig. 3e). All these results suggested that SIRT1 was the target gene of rno-miR-30c-5p.

### Silencing of SIRT1 reversed the effects of rno-miR-30c-5p inhibitor on the apoptosis and NF-κB pathway in IR myocardial cells

**Fig. 4:**
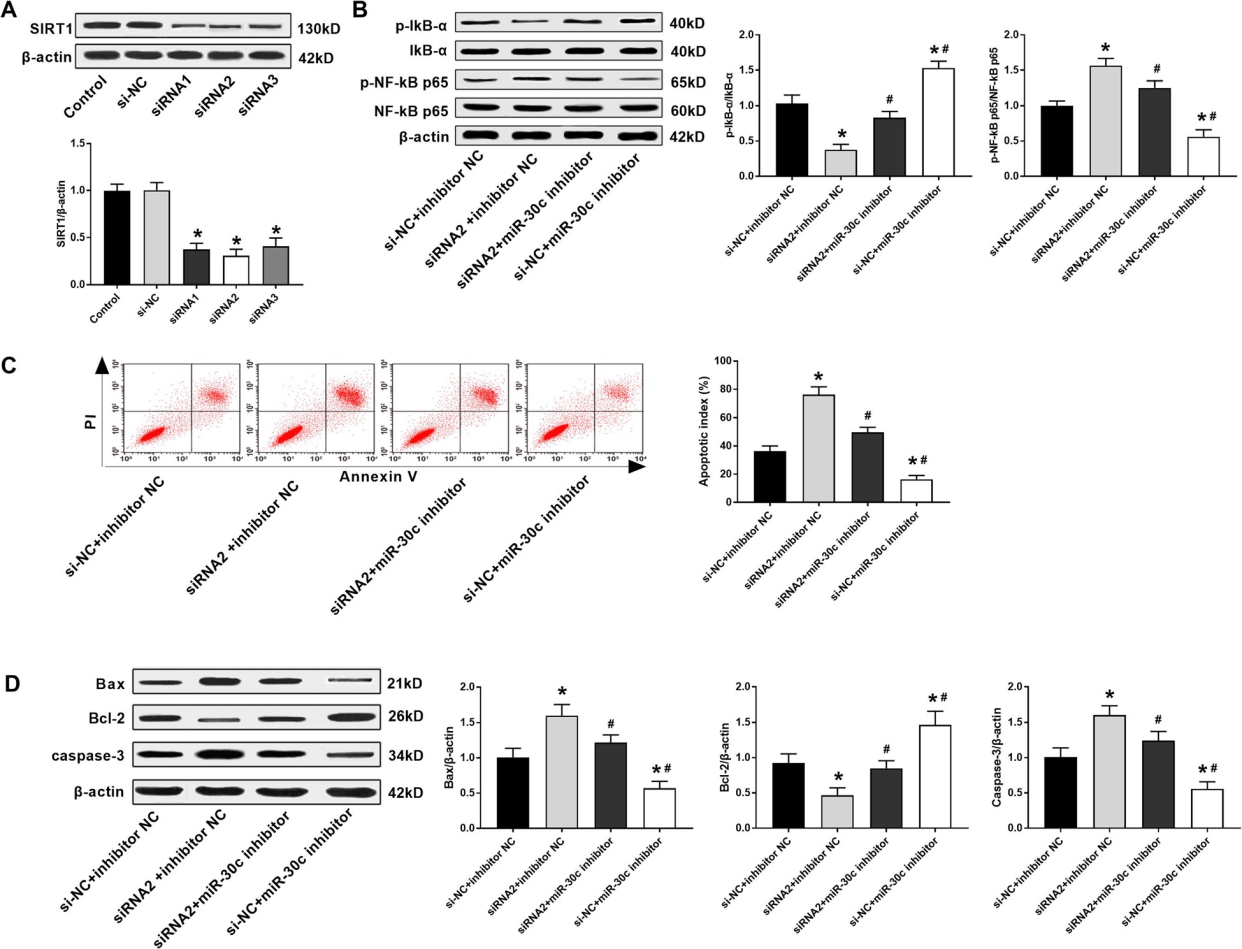
Rno-miR-30c-5p promoted the apoptosis, and activated NF-κB pathway in IR myocardial cells by targeting SIRT1. **b** The expression of SIRT1 was detected by Western blot (*N* = 3 each group). **b** The expression of p-IκBα, IκBα, p-NF-κB p65 and NF-κB p65 was detected by Western blot (*N* = 3 each group). **c** The apoptotic index (%) was measured by flow cytometry (*N* = 3 each group). **d** The expression of Bax, Bcl-2 and caspase-3 was detected by Western blot (*N* = 3 each group). Data were presented as mean ± SD (three independent repetitions for each sample). ^*^P < 0.05, vs. Control and si-NC group (**a**); ^*^P < 0.05, vs. si-NC + inhibitor NC group, ^#^*P* < 0.05, vs. siRNA2 + inhibitor NC group (**b-d**)

SIRT1 was silenced in IR myocardial cells by the transfection of siRNA1, 2 and 3. As shown in Fig. 4a, the protein expression of SIRT1 was significantly decreased by the transfection of siRNA1, 2 or 3 (*P* < 0.05). siRNA2 with relatively high silence efficiency was used for subsequent experiments. Compared with the si-NC + inhibitor NC group, the apoptotic index was significantly increased in the siRNA2 + inhibitor NC group, and was significantly decreased in the si-NC + rno-miR-30c-5p inhibitor group (*P* < 0.05). In addition, the protein expression of Bax, caspase-3, and p-NF-κB p65/NF-κB p65 was significantly increased in the siRNA2 + inhibitor NC group, and was significantly decreased in the si-NC + rno-miR-30c-5p inhibitor group compared with the si-NC + inhibitor NC group (*P* < 0.05). The protein expression of Bcl-2 and p-IκBα/IκBα was opposite to that of Bax in different groups (*P* < 0.05) (Fig. 4b and d). Note worthily, the effects of rno-miR-30c-5p inhibitor on the apoptosis and NF-κB pathway were reversed by the transfection of siRNA2 in IR myocardial cells (*P* < 0.05) (Fig. 4b-d). All these results suggested rno-miR-30c-5p could promote the apoptosis, and activated NF-κB pathway in IR myocardial cells by targeting SIRT1.

## Discussion

Myocardial infarction is one of the most common causes of death worldwide [19].

The therapeutic outcomes of patients receiving reperfusion are greatly limited by the occurrence of myocardial IR injury. It is urgent to explore the potential molecular mechanisms involving myocardial IR injury, and identify novel therapeutic targets. In this study, we demonstrated that rno-miR-30c-5p could promote the myocardial IR injury in rats through activating NF-κB pathway and down-regulating SIRT1.

Myocardial IR injury often leads to inflammation, and the inflammatory cascade reaction further induces the apoptosis of myocardial cells [20, 21]. MiRNAs exert important roles in myocardial I/R injury through regulating inflammation and cell apoptosis [22, 23]. For example, lentivirus expressing mmu-miR-146a attenuates I/R-induced myocardial apoptosis and inflammatory cytokine production in mice [18]. Intramyocardial injection of mmu-miR322 mimics diminishes cardiac apoptosis and reduces infarct size in IR mice [24]. Overexpression of rno-miR-144 significantly reduces the myocardial injury and apoptosis in IR rats [25]. Mmu-miR-24-3p decreases the infarct area and inhibits cell apoptosis in mice with myocardial IR injury. In this study, we found that the expression of rno-miR-30c-5p was significantly up-regulated in rats with myocardial IR injury. In vitro experiments confirmed that rno-miR-30c-5p enhances the inflammation and promotes the apoptosis of IR myocardial cells. Our findings indicate that rno-miR-30c-5p may enhance the myocardial IR injury via promoting inflammation and cell apoptosis. The promoting role of rno-miR-30c-5p on myocardial IR injury is consistent with that on I/R-induced kidney and spinal cord injury. Zhou et al. [13] have proved that ron-miR-30c-5p is up-regulated in rats with I/R-induced kidney injury. Li et al. [14] have shown that hydrogen sulfide protects spinal cord and induces autophagy in a rat model of spinal cord IR injury via down-regulating rno-miR-30c-5p. Silencing of ron-miR-30c-5p may be a potential therapeutic strategy for myocardial IR injury.

NF-κB is involved in the regulation of multiple biological processes including innate immunity, inflammation, cell proliferation and apoptosis [15, 16]. Under normal physiological condition, inactive NF-κB complexes are retained in the cytoplasm by binding to inhibitor of κB (IκB) proteins [26]. The stimuli can promote the phosphorylation and subsequent degradation of IκBα, and subsequently import the active NF-κB into the nucleus [26]. More and more studies have indicated that miRNAs exert vital roles in myocardial IR injury by regulating NF-κB pathway [9, 18, 27]. Li et al. [9] have confirmed that ron-miR-340-5p suppresses hypoxia/reoxygenation-induced apoptosis and oxidative stress in myocardial H9C2 cells via regulating Act1/NF-κB signaling. Liu et al. [27] have reported that the inhibition of mmu-miR-27a induces high thoracic epidural block to protect mice against myocardial IR injury via activating NF-κB pathway. In this study, overexpression and silencing of rno-miR-30c-5p significantly activated and blocked NF-κB pathway in IR myocardial cells. We speculate that rno-miR-30c-5p may promote the inflammation and apoptosis of myocardial cells in rats with myocardial IR injury through activating NF-κB pathway.

SIRT1 is a member of the sirtuin family that involved in the regulation of cell proliferation, apoptosis and autophagy [28, 29]. Emerging researches have indicated that SIRT1 is a potential therapeutic target for myocardial IR injury [30]. Yu et al. [31] have indicated that melatonin ameliorates IR-induced oxidative stress and endoplasmic reticulum stress via activating SIRT1 signaling in type 2 diabetic rats. Wang et al. [32] have proved that post-ischemic treatme

nt with lumbrokinase attenuates myocardial IR injury through the activation of Sirt1 signaling. Lin et al. [33] have demonstrated that the activation of SIRT1/Nrf2 signaling induced by Rutin contributes to the reduced oxidative stress and apoptosis of cardiomyocytes in rats with myocardial IR injury. Notably, a recent study showed that rno-miR-34a increases the apoptosis and infarct size and decreases left ventricular function through negatively regulating SIRT1 in rats with myocardial IR injury [34]. In this study, SIRT1 was identified as a target gene of rno-miR-30c-5p. We speculate that the up-regulation of SIRT1 may contribute to the promoting effect of rno-miR-30c-5p on myocardial IR injury. This speculation was further illustrated by that silencing of SIRT1 reversed the effects of rno-miR-30c-5p inhibitor on the apoptosis and NF-κB pathway in IR myocardial cells. Evidence has shown that SIRT1 inhibits the transcription of NF-κB through the deacetylation of NF-κB [35, 36]. The up-regulation of SIRT1 may relieve myocardial IR injury through blocking NF-κB signaling.

This study has some limitations. First, the regulatory role of rno-miR-30c-5p on myocardial IR injury is limited at the cellular level. The therapeutic effect of rno-miR-30c-5p silencing on rats with myocardial IR injury remains to be studied. Second, only rno-miR-30c-5p was studied. More miRNAs involving myocardial IR injury still need to be discovered based on microarray or RNA-seq methodologies. Third, only one target of rno-miR-30c-5p was selected. The discovery of more targets of rno-miR-30c-5p based on omics measurements is needed.

## Conclusions

In conclusion, rno-miR-30c-5p was up-regulated in rats with myocardial IR injury. Rno-miR-30c-5p enhanced the inflammation, promoted the apoptosis, and activated NF-κB pathway in IR myocardial cells through targeting SIRT1. Rno-miR-30c-5p may promote the myocardial IR injury in rats through activating NF-κB pathway and down-regulating SIRT1. Our research discovers a novel regulatory mechanism of rno-miR-30c-5p in myocardial IR injury and points out a novel therapeutic target.

## Supplementary information

**Additional File 1. Table S1.** miR-30-5p-all predicted transcripts

**Additional File 2. Table S2.** Predicted details of SIRT1R4

## Data Availability

The datasets used and/or analysed during the current study are available from the corresponding author on reasonable request. The genes analyzed in the present study are available at https://www.ncbi.nlm.nih.gov/search/ with Gene ID: 100314012 (microRNA-30c-5p, ENSMUSG00000065567; http://asia.ensembl.org/Mus_musculus/Gene/Summary?db=core;g=ENSMUSG00000065567;r=1:23291701-23291784;t=ENSMUST00000083633), and Gene ID: 309757 (Sirtuin 1, ENSMUSG00000020063; http://asia.ensembl.org/Mus_musculus/Gene/Summary?db=core;g=ENSMUSG00000020063;r=10:63319005-63381704).

## Abbreviations

LAD: Left anterior descending
mIRNAs: Micrornas
IR: Ischemia reperfusion
